# A novel mutation in the *GJA3* (connexin46) gene is associated with autosomal dominant congenital nuclear cataract in a Chinese family

**Published:** 2011-04-27

**Authors:** Guoxing Yang, Baogang Xing, Guangcai Liu, Xiangqing Lu, Xingang Jia, Xiangqing Lu, Xiuli Wang, Hongyan Yu, Yanjiang Fu, Jialiang Zhao

**Affiliations:** 1 Department of Opthalmology, Peking Union Medical College Hospital, Chinese Academy of Medical Sciences & Peking Union Medical College, Beijing, China; 2 Department of Opthalmology, Daqing eye Hospital, Heilongjiang, China

## Abstract

**Purpose:**

Congenital cataract is both a clinically and genetically heterogeneous lens disorder. The purpose of this study is to map and identify the mutation in an autosomal dominant congenital nuclear cataract in a Chinese family.

**Methods:**

Patients were given physical examinations and their blood samples were collected for DNA extraction. Genotyping was performed by microsatellite markers and logarithm of odds (LOD) scores were calculated using the LINKAGE programs. Mutation detection was performed by direct sequencing.

**Results:**

Linkage to the gap-junction protein α3 (*GJA3*) locus was verified. Sequencing of *GJA3* revealed a G>A transition at nucleotide position c.139, which causes an Asn substitution for the conservative Asp at codon 47 (P.D47N).This mutation is identified in all affected individuals but is not found in 100 control chromosomes.

**Conclusions:**

Our results identify that the mutation (D47N) in GJA3 is responsible for this Chinese pedigree. It is further identified that *GJA3* is responsible for congenital cataract. In our study, the novel mutation broadens the spectrum of *GJA3* mutations.

## Introduction

Congenital cataract is a significant cause of vision loss, causing approximately one third of blindness in infants. Congenital cataract occurs in approximately 1–6/10,000 of live births. One quarter of congenital cataract is hereditary [1-4]. Hereditary (Mendelian) cataract is mostly inherited as autosomal dominant (AD) mode of transmission, but X-linked and autosomal recessive forms were also observed. Congenital cataract is a clinically and genetically heterogeneous lens disorder. Phenotypically identical cataracts can be resulted from mutations at different genetic loci and can have different inheritance patterns. While in a same genetic locus or a single large family, phenotypically different cataract types also can be found. To date, about forty genetic loci have been shown to be linked with congenital cataract and twenty-six genes have been cloned [5], including crystallins, connexins, heat shock transcription factor-4, aquaporin-0, and beaded filament structural protein-2. Types of mutation and the morphology of cataract are believed to be related [5].

Recently, we found a four-generation pedigree with congenital nuclear cataract from Northeast China. Linkage to the gap-junction protein α3 (*GJA3*) locus was verified. Mutation screening in *GJA3* identified a G→A transition at nucleotide position c.139. This nucleotide change resulted in an Asn substitution for the highly conserved Asp at codon 47 (P.D47N). The mutation was only cosegregated in the patients and was not found in the 100 control chromosomes.

## Methods

### Patients and clinical data

The four-generation family enrolled in this study was found in a Northeastern providence of China. Clinical examination, peripheral blood collection, and DNA extraction were performed in the Department of Ophthalmology, Peking Union Medical College Hospital, Beijing, China. Informed consent in accordance with the Declaration of Helsinki and the Institutional Review Board and Ethics Committee of Peking city was obtained from all participants. The family includes twenty-two conformed patients with congenital nuclear cataract. Clinical data of these subjects was ascertained by detailed ocular examinations.

### Genotyping and linkage analysis

With fluorescently labeled microsatellite markers, linkage analysis of candidate gene region at 13q was done on the DNA samples from fifteen ophthalmologically examined individuals. Two-point linkage analysis was performed by MLINK from LINKAGE Program Package.

### Mutation analysis

Coding exons of *GJA3* were amplified by polymerase chain reaction (PCR) using a set of three pairs of primers (Table 1) [6]. The PCR products were sequenced on an ABI3730 Automated Sequencer (PE Biosystems, Foster City, CA).

**Table 1:** Primers used for *GJA3* amplification.

**Exon**	**primer (5′-3′)**	**Product length (bp)**	**Annealing temperature (°C)**
*GJA3*-1F	CGGTGTTCATGAGCATTTTC		
*GJA3*-1R	GACGTAGGTCCGCAGCAG	496	58
*GJA3*-2F	GCAGGACAATCCCTCGTC		
*GJA3*-2R	GGTCAGGGCTAGCAGTTTGA	532	58
*GJA3*-3F	TCGGGTTCCCACCCTACTAT		
*GJA3*-3R	TGCACTTTGGTTTTGGTTTC	579	58

Cited from [6].

## Results

### Clinical findings

**Figure 1:**
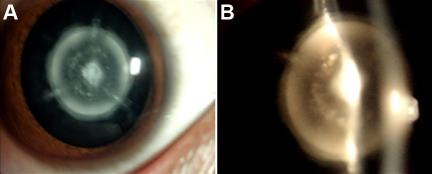
Slit lamp photograph showing nuclear cataract of patients IV:7 (**A**, **B**; in Figure 2).

We verified a four-generation family with twenty-two confirmed individuals with congenital cataract (Figure 1). The affected individuals presented with bilateral congenital nuclear cataracts that consisted of a central nuclear opacity affecting the embryonic and fetal nucleus of the lens. Blood samples were collected from ten of the twenty-two patients and five normal members. All participated patients have no other clinical related ophthalmic syndromes.

### Linkage and haplotype analysis

**Figure 2:**
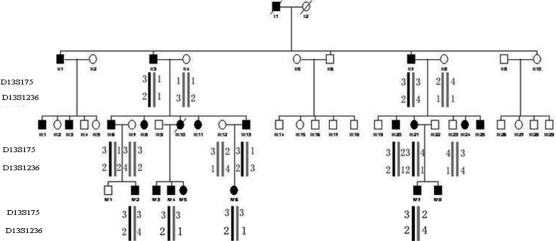
Pedigree and haplotype of the family. A four-generation pedigree with fifteen available members is shown. Haplotype analysis of the family demonstrated segregation of two microsatellite markers on chromosome 13q.

We obtained positive logarithm of odds (LOD) scores at 13q (Table 2). A maximum positive LOD score of 2.055 at θ=0.000 was obtained by marker D13S1236. The haplotype showed complete cosegregation in all the 10 analyzed affected individuals (Figure 2).

**Table 2:** Results of linkage analysis.

** **	**LOD**	**Scores**	**At θ=**			
Marker	0.000	0.010	0.100	0.200	0.300	0.400
D13S175	2.008	1.971	1.626	1.215	0.786	0.370
D13S1236	2.055	2.015	1.649	1.215	0.758	0.313

### Mutation analysis

**Figure 3:**
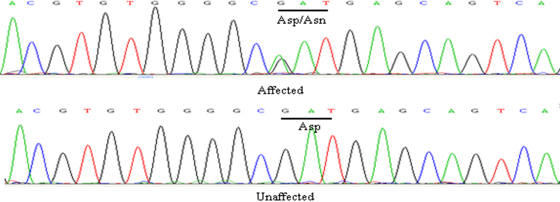
DNA sequences of *GJA3* in unaffected and affected individuals. A heterozygous change G→A at nucleotide position 139, resulting in the substitution of aspartic acid by asparagine (D47N) in the affected individuals.

**Figure 4:**
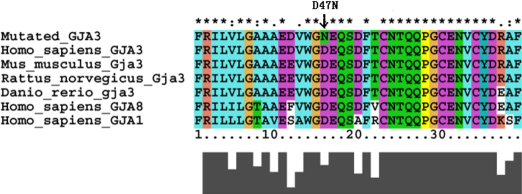
A multiple sequence alignment of amino acid sequence of GJA3 (connexin46) in different species and in different human α-connexins. The Asp47 residue is highly conserved during evolution.

By direct sequencing of the *GJA3* coding region, a novel heterozygous G→A transition at nucleotide position 139 (c. 139G>A) was detected in this family (Figure 3). This transition leads to the replacement of a highly conserved Asp to Asn at codon 47 (P.D47N; Figure 4). The mutation was only cosegregated in the patients and was not found in other normal members and the 100 control chromosomes from the same ethnic background.

## Discussion

The intercellular gap junctions allow the flow of ions, second messengers, and metabolites, which preserves homeostasis in lens fiber cell. Structural proteins belonging to the connexin multigene family make up the intercellular channels presented in gap junctions. Three distinct connexin (CX) genes (Connexin43 [*CX43*], Connexin46 [*CX46*], and Connexin50 [*CX50*]) are expressed in the lens [7].

So far, all the families with *CX46* or *CX50* mutations have the same phenotype (nuclear), confirming that these transport membrane proteins, despite of their low-level expressions, are important for the correct embryological development of the lens [8].

Six connexin (Cx) subunits can form a hemi-channel (connexon) in the plasma membrane. It can dock to another hemi-channel in the plasma membrane of another cell nearby, to assemble a complete gap junctional channel. Cell adhesion proteins may contribute to the docking and assembling process. The connexin subunit is a four transmembrane spanning protein, harboring two extracellular domains (E1, E2), one cytoplasmic loop and one cytoplasmatic NH_2_- as well as COOH-terminal region. Both the E1 and E2 domains are believed to function in the docking procedure via cysteine-cysteine disulfide bridges in intercellular space, forming the gap-junction channels (connexin dodecamers). As a result, mutations in these extracellular domains may impair Cx46 mediated coupling of lens fiber cells, and trigger lens opacities [7].

In this pedigree, the mutation D47N in the E1 domain of *GJA3* may influence the hemi-channel docking and subsequently cause congenital cataract. In the previous studies, three mutations (W45S, P59L, and N63S) in the E1 domain of *GJA3* have been proved to be associated with dominant congenital cataract [6,9,10].

Both of Cx46 and Cx50 co-localize in gap junctions and assemble the heteromeric channel to maintain the lens clarity. Both the connexins are associated with congenital cataracts of humans. According to the similarity of protein sequence, structure and function of Cx46 and Cx50, the codon 47 of Cx46 and Cx50 are conserved in human and mouse. Previous studies illustrated that congenital cataract was associated with D47A (in the *No2* mouse) and D47Y (in human) of E1 domain in Cx50 [11-13].

In conclusion, we find a heterozygous G→A transition at nucleotide position c.139 in *GJA3* from an autosomal dominant congenital nuclear cataract family. These findings thus expand the mutation spectrum of *GJA3*.
